# Adolescent Alcohol Exposure Results in Sex-specific Alterations in Conditioned Fear Learning and Memory in Adulthood

**DOI:** 10.3389/fphar.2022.837657

**Published:** 2022-02-08

**Authors:** L. Judson Chandler, Dylan T. Vaughan, Justin T. Gass

**Affiliations:** ^1^ Department of Neuroscience, Medical University of South Carolina, Charleston, SC, United States; ^2^ Department of Biomedical Sciences, East Tennessee State University, Johnson City, TN, United States

**Keywords:** fear conditioning, fear extinction, fear recovery, freezing, adolescent alcohol exposure, mGlu5, sex differences

## Abstract

The present study used auditory fear conditioning to assess the impact of repeated binge-like episodes of alcohol exposure during adolescence on conditioned fear in adulthood. Male and female Long-Evans rats were subjected to adolescent intermittent ethanol (AIE) exposure by vapor inhalation between post-natal day 28 and 44. After aging into adulthood, rats then underwent fear conditioning by exposure to a series of tone-shock pairings. This was followed by cued-tone extinction training, and then testing of fear recovery. In male rats, AIE exposure enhanced conditioned freezing but did not alter the time-course of extinction of cued-tone freezing. During subsequent assessment of fear recovery, AIE exposed rats exhibited less freezing during contextual fear renewal, but greater freezing during extinction recall and spontaneous recovery. Compared to males, female rats exhibited significantly lower levels of freezing during fear conditioning, more rapid extinction of freezing behavior, and significantly lower levels of freezing during the tests of fear recovery. Unlike males that were all classified as high conditioners; female rats could be parsed into either a high or low conditioning group. However, irrespective of their level of conditioned freezing, both the high and low conditioning groups of female rats exhibited rapid extinction of conditioned freezing behavior and comparatively low levels of freezing in tests of fear recovery. Regardless of group classification, AIE had no effect on freezing behavior in female rats during acquisition, extinction, or fear recovery. Lastly, exposure of male rats to the mGlu5 positive allosteric modulator CDPPB prevented AIE-induced alterations in freezing. Taken together, these observations demonstrate sex-specific changes in conditioned fear behaviors that are reversible by pharmacological interventions that target mGlu5 receptor activation.

## Introduction

Formation of emotional memories are an important mechanism for how animals, including humans, learn to avoid threatening and aversive environmental stimuli. While fear learning and memory is typically a healthy process, it can also be maladaptive and pathological, and is an important etiological factor in many psychological disorders. In animal studies, Pavlovian auditory fear conditioning is a leading model for investigating associative fear learning and memory. In this paradigm, a neutral stimulus (auditory tone) is temporally paired with an aversive stimulus (e.g. a foot shock) such that repeated tone-shock pairings lead to a learned association. Subsequent presentation of the conditioned stimulus (CS) alone elicits a defensive response (e.g. freezing) even without presentation of the unconditioned stimulus (US). However, when the CS is repeatedly presented without being paired with the US, the conditioned response can be extinguished. Extinction is not simply the unlearning or forgetting of the original associative fear memory, but instead involves the formation a new inhibitory memory (Tovote et al., 2015; Bouton et al., 2021). Importantly, while this inhibitory extinction memory may initially dominate, the original fear memory remains and can subsequently be reactivated in response to environmental context and cues.

Adolescence is characterized as a period of increased impulsivity, risk-taking and sensation seeking, and as such is also a time when many individuals begin experimenting with alcohol (Spear, 2018; Crews et al., 2019). Of particular concern is that alcohol consumption in this age group frequently occurs in repeated episodes of “extreme” binge drinking that result in high levels of intoxication. For example, a recent national survey of adolescent youths revealed that 4.5% of 8th graders and 16.8% of 12th graders reported having engaged in “extreme” binge drinking, which was defined as having consumed 10 or more drinks in a single session (Patrick and Terry-McElrath, 2019). Adolescence is also a period of continued brain remodeling and rapid behavioral development that includes maturation of top-down executive control over behavior (Casey et al., 2008; Drzewiecki and Juraska, 2020).

While the developing adolescent brain is highly plastic, this feature may also enhance its vulnerability to adverse environmental insults such as those associated with repeated episodes of extreme binge intoxication that can produce long-lasting changes in brain structure, function, and behavior. In support of this, recent studies in rodent models of adolescent alcohol binge intoxication have reported a number of deficits in behavioral control and decision-making in adulthood that are consistent with a general reduction of behavioral flexibility and adaptive decision-making (Spear, 2018). Changes that have been reported include deficits in set-shifting (Gass et al., 2014a; Fernandez and Savage, 2018), reversal learning (Coleman et al., 2014), habitual behaviors (Barker et al., 2017; Towner and Spear, 2021), increases in risky decision-making (Clark et al., 2012), resistance to extinction of ethanol self-administration (Gass et al., 2014b), and increases in anxiety-like behaviors (Pandey et al., 2015). Interestingly, it has become clear in recent years that many of the fundamental principles of learning and memory also play a major role in the development and maintenance of deficits associated with addictions and trauma and anxiety-related disorders (Tipps et al., 2014). Previous findings from our lab have shown that pharmacological manipulation of the metabotropic glutamate receptor 5 (mGlu5) can attenuate behavioral deficits associated with chronic alcohol and stress (Gass et al., 2014a; Smiley et al., 2020; Smiley et al., 2021a). However, the impact of adolescent alcohol exposure on fear learning in adulthood has not been widely investigated, and even less is known regarding sex-specific differences as most previous studies have been conducted in males. It is of particular interest that many of the key brain regions and neurocircuits implicated in different aspects of monitoring of threats (Bouton et al., 2021) also undergo important developmental changes during adolescence, and thus may be particularly vulnerable to repeated episodes of excessive ethanol intoxication during adolescence. Therefore, the present study utilized a well-characterized model of binge-like alcohol exposure and a Pavlovian fear conditioning paradigm to determine the impact of adolescent alcohol exposure on fear learning and memory in adult male and female rats. An additional aim of this study was to examine the ability of mGlu5 modulation to prevent or attenuate deficits in fear-related behaviors following chronic alcohol exposure during the adolescent period.

## Materials and Methods

### Animals

Long-Evans dams, shipped with 10 pups of both sexes that were postnatal day (PD) 22 upon arrival, were obtained from Envigo (Indianapolis, IN). At PD 24, pups were weaned and pair-housed in standard polycarbonate cages. The vivarium was maintained on a reverse 12:12 light-dark cycle with lights off at 09:00. All rats were provided with Teklad 2918 (Envigo) standard chow and water *ad libitum*, and all experimental procedures were conducted with the approval of the Institutional Animal Care and Use Committee at the Medical University of South Carolina and adhered to the guidelines set forth by the National Research Council’s Guide for the Care and Use of Laboratory Animals (NRC, 2011).

### Drugs

3-cyano-N-(1,3-diphenyl-1H-pyrazol-5-yl) benzamide (CDPPB) was custom synthesized by Chemir Analytical Services (Maryland Heights, MO) according to previously published methods (Lindsley et al., 2004; Kinney et al., 2005), purified to >95% purity by liquid chromatography-mass spectrometry, and suspended in 10% v/v Tween-80 (Sigma-Aldrich). CDPPB was administered as a subcutaneous injection at a dose of 30 mg/kg, which was based on studies showing that a 30 mg/kg dose of CDPPB can facilitate learning and recover behavioral deficits without resulting in any motor effects (Gass and Olive, 2009; Cleva et al., 2011; Gass et al., 2014b; Gass et al., 2014b). All control groups received an injection with vehicle only (10% Tween 80) using the same dosing regimen. Additionally, the half-life of CDPPB is approximately 4.4 h (Kinney et al., 2005).

### Adolescent Intermittent Ethanol Exposure

**FIGURE 1:**
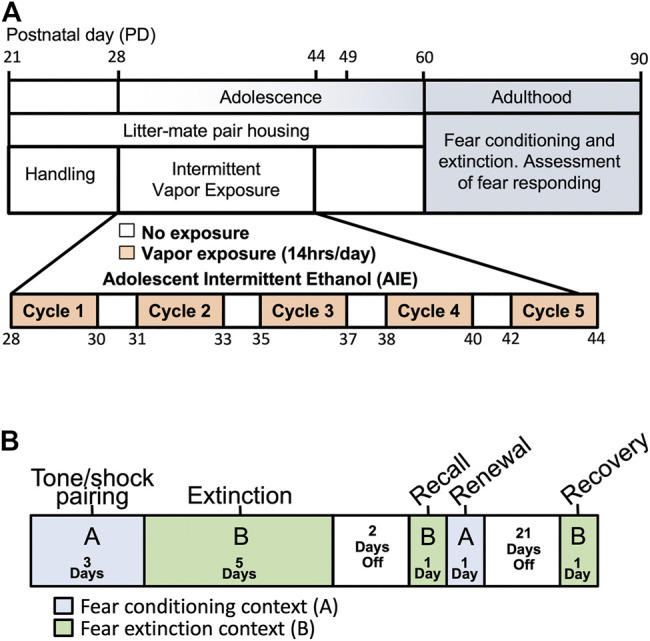
Schema of experimental time-line and procedures. **(A)** Pair-housed adolescent male and female rats were subjected to 5 cycles of either Air (control) or ethanol vapor beginning at PD28 and ending at PD44. Rats were then allowed to age to adulthood when behavioral testing was initiated. **(B)** An ABBAB contextual design was used to examine fear conditioning, extinction, recall, renewal and spontaneous recovery.

Adolescent intermittent ethanol (AIE) exposure was carried out via vapor inhalation as previously described (Gass et al., 2014a; Nentwig et al., 2019). In brief, each set of pair-housed rats were randomly assigned to an AIE or AIR control group, with rats from each litter split evenly across groups. As illustrated in Figure 1A, adolescent rats were subjected to 5 cycles of Air or ethanol vapor between PD 28–44, with each cycle consisting of two consecutive days of intermittent exposure to ethanol vapor followed by two ethanol-free days. The litter-matched control animals were treated identically except they were not exposed to ethanol vapor. Each vapor exposure day began at 18:00 and lasted for 14 h. AIE exposed rats were scored for behavioral signs of intoxication immediately upon removal from the vapor chambers. This involved scoring motoric behavior according to the following 5 point scale: 1 = No signs of intoxication; 2 = Slightly intoxicated (slight motor impairment); 3 = Moderately intoxicated (obvious motor impairment but able to walk); 4 = Highly intoxicated (dragging abdomen, loss of righting reflex); 5 = Extremely intoxicated (loss of righting reflex and loss of eye blink reflex). Tail-vein blood samples were collected after the second exposure day of each cycle for determination of blood ethanol concentrations (BECs) using an Analox alcohol analyzer (Analox Instruments, Atlanta, GA). Following completion of the last exposure cycle, rats were left undisturbed in their home cages until individually housed at PD 60.

### Fear Conditioning, Extinction, and Recovery

Rats were tested using a fear conditioning, extinction and recovery paradigm that is based on previously published studies (Smiley et al., 2020; Smiley et al., 2021b). As is depicted in Figure 1B, an “ABBAB” contextual design was used to assess conditioned fear acquisition, cued fear extinction, extinction recall (retention), context-dependent fear renewal, and spontaneous recovery (Cain et al., 2012; Vanelzakker et al., 2013). In this ABBAB timeline, initial fear conditioning occurred in context A, cued-fear extinction occurred in a novel context B (consisting of differently colored walls and grooved plexiglass flooring). Following extinction training, recall of the extinction memory was assessed in the context B environment, contextual renewal of the original fear memory was assessed by placing the rats back in the original conditioning environment (context A). Finally, spontaneous recovery was assessed 21 days later in context B extinction environment.

The fear conditioning phase consisted of a 120 s acclimation period followed by three pairings of the CS (30 s, 80 dB, 3 kHz tone) and the US (2 s, 0.75 mA scrambled foot-shock) in which the US is presented during the last 2-s of the CS. A single 5-min session occurred daily for 3 consecutive days. The extinction training phase consisted of a 120 s acclimation period followed by 10 presentations of the CS (tone), each lasting 30 s and separated by a 10 s no-stimulus interval. Rats receive 5 days of cue extinction training consisting of 10 trials per day. For examination of the effects of mGlu5 positive allosteric modulation (PAM) on fear conditioning and extinction, CDPPB (30 mg/kg, s.c) was administered each day 20 min prior to initiation of each extinction session. Two days after completion of extinction training, rats were tested for extinction recall by placing them into the cue extinction environment (context B) and a single presentation of the tone. The following day, rats were placed back into the conditioning environment (context A) to assess context-dependent renewal of the fear memory. To assess spontaneous recovery of the extinction memory 21 days after the end of extinction training, freezing behavior in context B was measured in response to presentation of a single tone. In all phases of the task, freezing behavior was determined from digitized videos using FreezeScan (Clever Systems, Inc) as previously described (Quirk and Mueller, 2008; Quirk et al., 2010; Sierra-Mercado et al., 2011; Milad and Quirk, 2012).

### Statistical Analysis

All data sets were analyzed using analysis of variance (ANOVA) with a Sidak post-hoc comparisons test. All statistical analysis was performed in Prism 9.0. Effects were considered statistically significant at *p* ≤ 0.05.

## Results

The goal of the present study was to determine the effects of repeated episodes of alcohol intoxication during adolescence on fear conditioning, extinction, and recovery in adulthood. The experimental approach employed a model of intermittent alcohol exposure by vapor inhalation that is designed to simulate repeat episodes of binge-like alcohol exposure. The procedure involved subjecting adolescent rats to 5 intermittent cycles of ethanol vapor between PD 28–44, a period that encompasses the early to middle stage of adolescence. Behavioral intoxication and blood ethanol concentration (BEC) were assessed at the end of each of the 5 exposure cycles. The average intoxication scores using the 1-5 point rating scale was 2.66 ± 0.16 for male rats and 2.4 ± 0.09 for female rats, which represents a moderate level of intoxication on the rating scale. The corresponding BEC values were 375 ± 9.6 mg/dl for the male rats and 271 ± 12.7 mg/dl for the female rats. Unpaired t-test comparisons indicated the BEC levels in the males was significantly higher than in the females (*p* < 0.0001).

**FIGURE 2:**
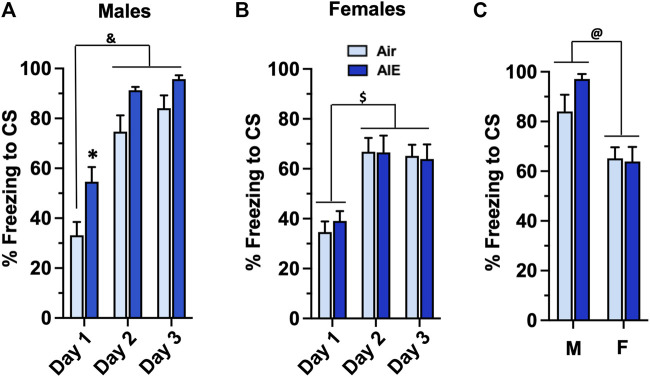
AIE exposure results in sex-specific increases in the acquisition of conditioned fear in adulthood. **(A)** Adult male rats that had undergone adolescent intermittent ethanol (AIE) exposure exhibited a significant increase in freezing during tone-shock pairings compared to male Air control rats. **(B)** In contrast, adult female rats that had undergone AIE exposure did not exhibit any differences in freezing during the tone-shock pairings compared to the female Air control rats. **(C)** Comparison of percent freezing on Conditioning Day 3 revealed that female rats exhibited significantly lower levels of freezing compared to male rats. *, significant difference from respective Day 1 Air control, (*p* = 0.0295); &, significant difference of Day 1 and 2 groups compared to Day 1 Air control (all *p* values < 0.0001); $, significant difference of Day 1 groups compared to Day 2 and 3 groups (all *p* values < 0.01); @, significant group difference of male compared to female groups (*p* = 0.0001); *n* = 14/group for males, 16/group for females.

To determine whether AIE impacted conditioned fear behaviors assessed during adulthood, the Air control and AIE exposed adult rats were subjected to 3 consecutive days of tone-shock pairing. For male rats (Figure 2A), a two-way ANOVA revealed a significant main effect of Conditioning Day (F (2, 78) = 54.73, *p* < 0.0001) and treatment (AIE vs Air) (F (1, 78) = 18.28, *p* < 0.0001). Sidak’s post-hoc analysis revealed a significant difference of Air control rats at Conditioning Day 1 compared to all groups on Conditioning Day 2 and 3 (all *p* values < 0.0001). Post-hoc analysis also revealed a significant increase of freezing in the AIE rats compared to the Air control rats at Conditioning Day 1 (*p* = 0.0295). For female rats (Figure 2B), ANOVA revealed a significant main effect of Conditioning Day (F (2, 102) = 20.23, *p* < 0.0001). Sidak’s post-hoc test indicated that freezing by both Air and AIE rats at Condition Day 1 was significantly lower than freezing at Conditioning Days 2 and 3 (all *p* values < 0.01). The ANOVA also indicated that AIE had no effect on freezing in the female rats (F (1, 102) = 0.05625, *p* = 0.8130). To determine if there was a significant sex difference in the acquisition of conditioned fear, we next performed an ANOVA comparing freezing at Conditioning Day 3 for the male versus female rats. As shown in Figure 2C, this revealed that male rats froze significantly more than their female counterparts (F (1, 48) = 18.41, *p* < 0.0001).

**FIGURE 3:**
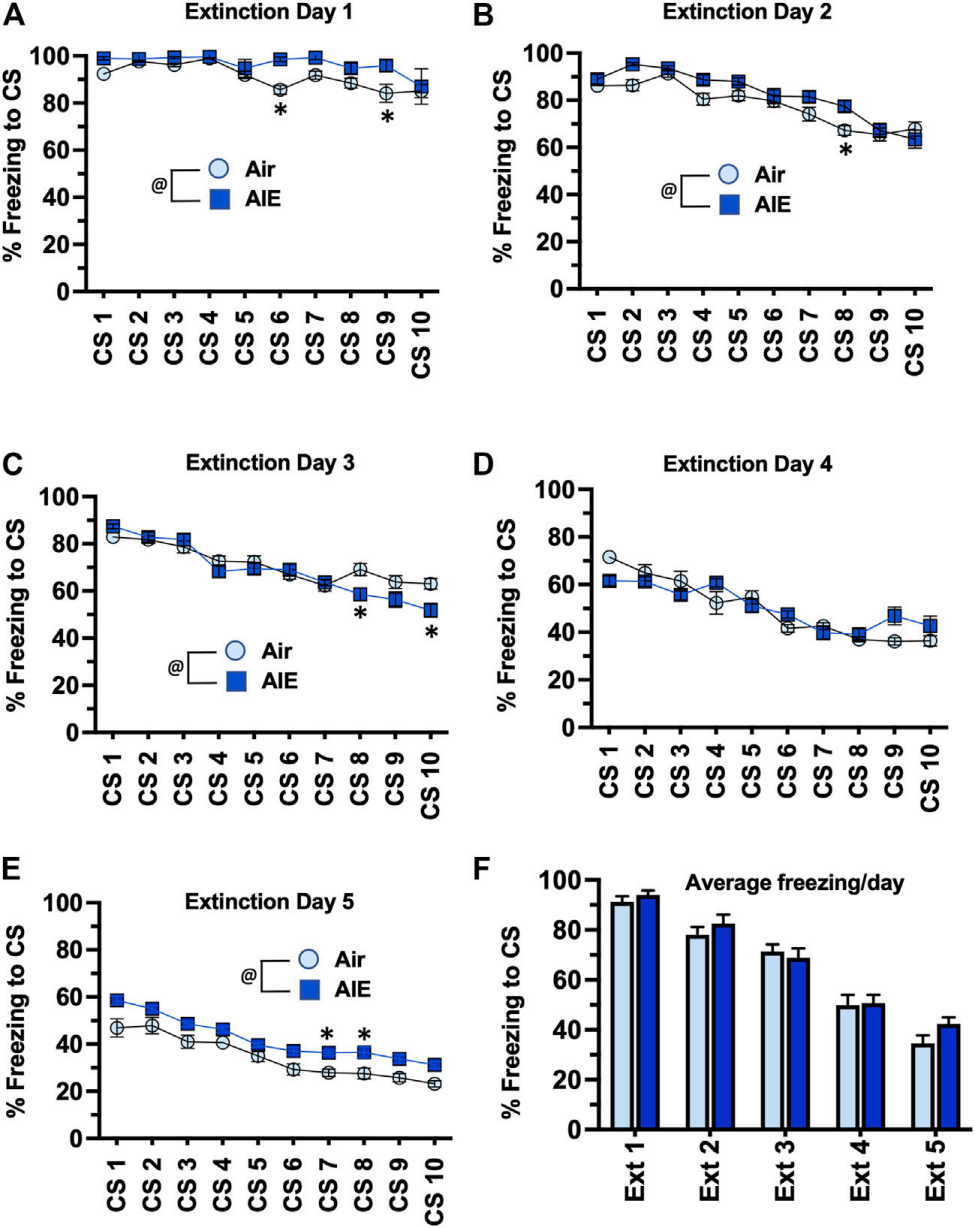
AIE exposure did not alter extinction of conditioned fear in adult male rats. **(A–E)** Five consecutive days of presentation of the conditioned tone without the shock (10/session/day) resulted in a day and session-dependent reduction of freezing to the conditioned stimulus. There were small but statistically significant different increases and decreases in the levels of freezing in the AIE versus Air control rats that depended upon the particular day of extinction. However, comparison of the average freezing across the 10 tones for each daily extinction session **(F)** revealed there were no significant differences between the Air control and AIE exposed rats at any of the 5 extinction days. @, significant group difference of Air versus AIE (all *p* values < 0.05); *, significant difference compared to respective CS tone # (all *p* values < 0.05); *n* = 14/group.

**FIGURE 4:**
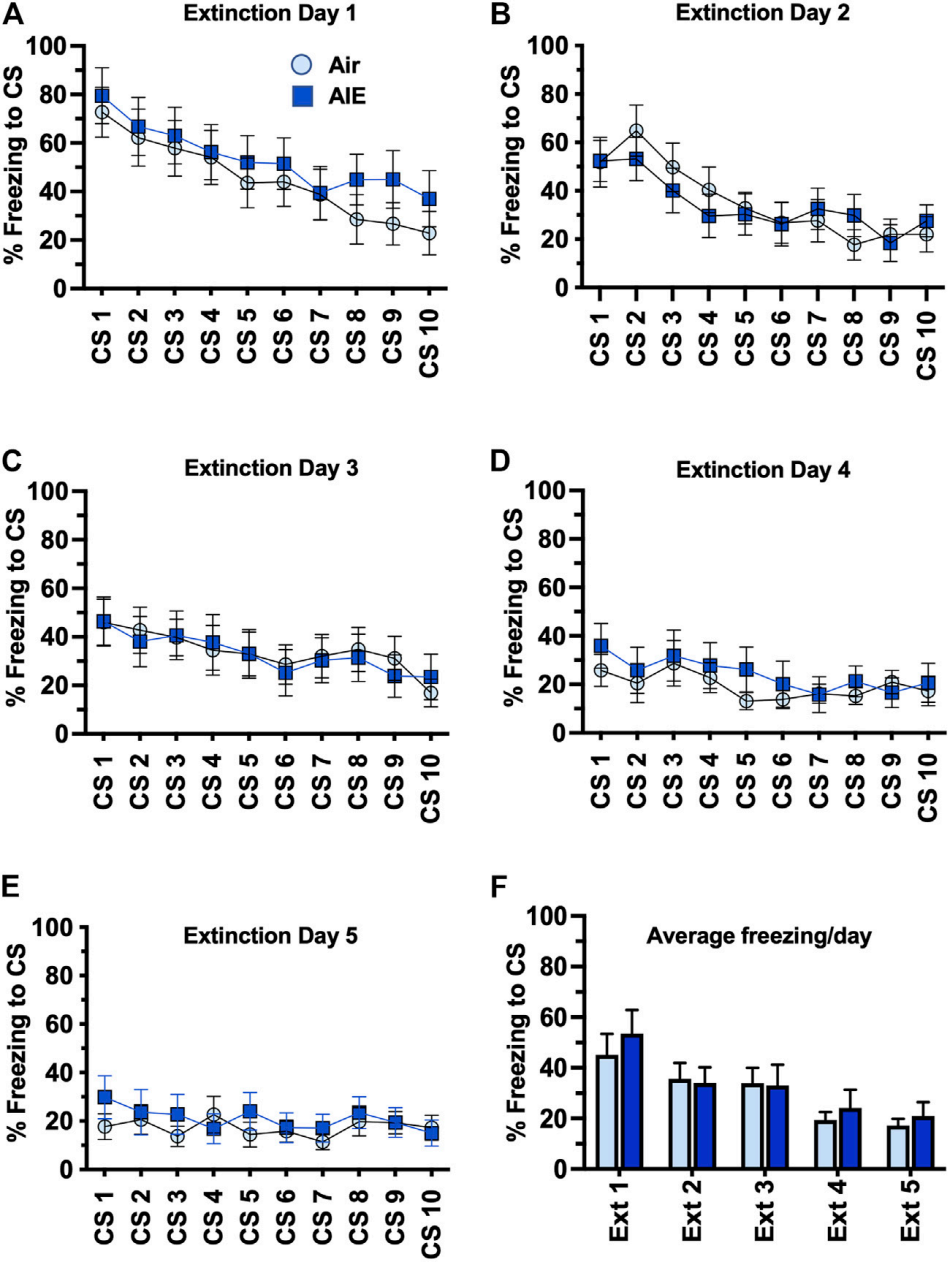
AIE exposure did not alter extinction of conditioned fear in adult female rats. **(A–E)** Five consecutive days of presentation of the conditioned tone without the shock (10/session/day) resulted in a day and session-dependent reduction of freezing to the conditioned stimulus, but there was no effect of AIE. **(F)** Comparison of the average freezing across the 10 tones of each daily extinction session further confirmed there was no significant difference between the Air control and AIE exposed rats. *n* = 16/group.

Following completion of tone-shock fear conditioning, rats were subjected to 5 daily sessions of fear extinction in a novel environment that involved 10 presentations of the CS (tone) in the absence of the US (shock). As shown in Figures 3A–E, ANOVA of freezing during fear extinction by male rats revealed a day by session-dependent reduction in freezing behavior as evidenced by a main effect of the number of CS (CS#) presentations {Day 1: F (9, 260) = 5.504, *p* < 0.0001; Day 2: F (9, 260) = 31.94, *p* < 0.0001; Day 3: F (9, 260) = 34.38, *p* < 0.0001; Day 4: F (9, 260) = 29.70, *p* < 0.0001; Day 5: F (9, 260) = 39.23, *p* < 0.0001}. There was also a significant main effect of AIE at all extinction days except Day 4 {Day 1: F (1, 78) = 18.28, *p* < 0.0001; Day 2: F (1, 260) = 17.68, *p* < 0.0001; Day 3: F (1, 260) = 5.494, *p* = 0.019; Day 5: F (1, 260) = 71.24, *p* < 0.0001}. Post-hoc analysis also revealed both increases and decreases in freezing in response to the CS that depended on the day of extinction (all *p* values < 0.05). However, while comparison of the average freezing across the 10 CS for each extinction day (Figure 3F) indicated there was a significant main effect of Extinction Day (F (4, 130) = 98.80, *p* < 0.0001), there was no main effect of treatment (Air vs AIE) (F (1, 130) = 1.838, *p* = 0.1775). For fear extinction in female rats (Figures 4A–E), there was a significant effect of CS# on Extinction Day 1 (F (9, 340) = 3.652, *p* = 0.0002) and Day 2 (F (9, 340) = 4.657, *p* < 0.0001), but no effect of AIE on any of the extinction days. Comparison of the average freezing across the 10 CS of each extinction day (Figure 4F) confirmed there was a significant effect of Extinction Day (F (4, 170) = 6.605, *p* < 0.0001), but no effect of treatment (F (1, 170) = 0.4692, *p* = 0.4943).

**FIGURE 5:**
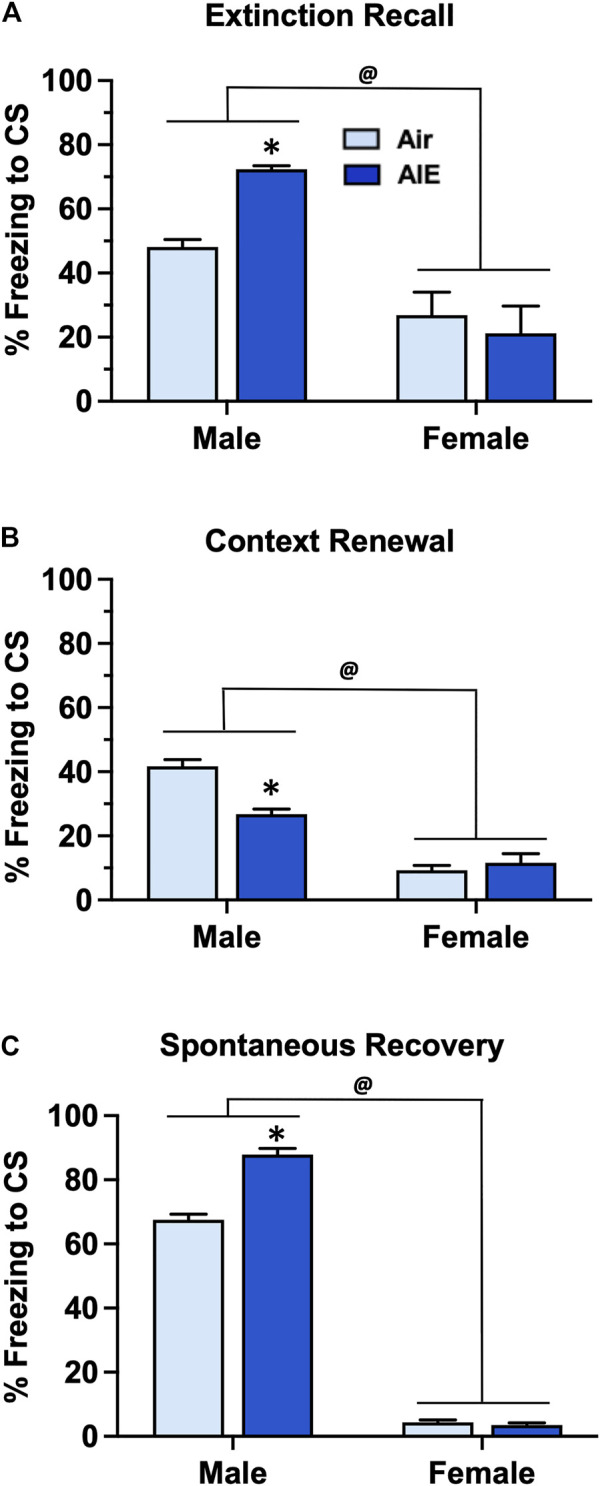
AIE exposure resulted in sex-specific alterations in fear recall, renewal and spontaneous recovery. **(A)** Following 5 days of fear extinction training, Air control and AIE exposed adult male and female rats were tested for extinction recall by assessing freezing behavior in response to exposure to a single conditioned tone. In response to tone presentation, male rats exhibited significantly higher levels of freezing compared to their female counterparts. AIE exposed male rats also exhibited significant increases in freezing during recall compared to the male Air controls. In contrast, AIE had no effect on recall in female rats. **(B)** Following testing of recall, male and female rats were tested for contextual fear renewal by assessing freezing behavior in response to re-exposure to the original conditioning context. Female rats exhibited significantly lower levels of freezing in response to re-exposure to the conditioning context compared to male rats. AIE exposed male rats also exhibited significant decreases in context-dependent freezing compared to the male Air control rats. AIE had no effect on contextual renewal in female rats. **(C)** Rats were tested for spontaneous recovery 21 days after the last extinction session by placing them back into the extinction environment. Female rats exhibited significantly lower levers of freezing in response to re-exposure to the extinction environment compared to the male rats. AIE exposed male rats also exhibited significant increases in spontaneous recovery compared to the male Air control rats. AIE had no effect on spontaneous recovery in female rats. *, significant difference of AIE versus respective Air control group; all *p* values < 0.05. @, significant difference of male compared to female rats; all *p* values < 0.0001; *n* = 14/group for males, 16/group for females.

Following completion of extinction training, animals were sequentially tested for fear recall, context renewal, and spontaneous recovery 21 days after completion of extinction training. For recall (Figure 5A) ANOVA revealed that female rats exhibited significantly lower levels of freezing compared to male rats (F (1, 60) = 31.88, *p* < 0.0001). Post-hoc analysis indicated that AIE exposed male rats froze significantly more than the corresponding male Air controls (*p* = 0.0288). With context renewal (Figure 5B), female rats again exhibited significantly lower levels of freezing compared to the male rats (F (1, 60) = 125.4, *p* < 0.0001). Post-hoc analysis also indicated that male AIE rats froze significantly less than male Air control rats (*p* < 0.0001). For spontaneous recovery (Figure 5C), ANOVA also indicated that female rats froze significantly less than male rats (F (1, 60) = 3296, *p* < 0.0001). Post-hoc analysis further revealed that AIE significantly increased freezing in the male rats (*p* < 0.0001) but not female rats.

**FIGURE 6:**
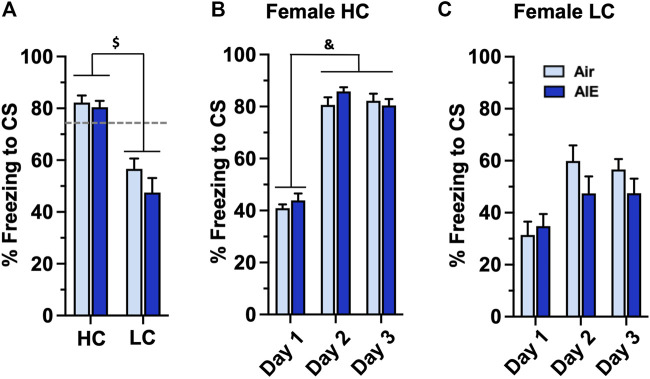
AIE exposure did not alter the acquisition of conditioned fear in either high conditioning (HC) or low conditioning (LC) female rats. Female rats were classified as either high or low conditioning rats using a cut-off criteria based upon the minimal level of conditioning exhibited by male rats. **(A)** Comparison of percentage of freezing at conditioning day 3 revealed a significant difference in the level of freezing between the HC and LC groups. Examination of the time-course of freezing across conditioning for the HC **(B)** and LC **(C)** female rats revealed that the Air control and AIE exposed rats in each group conditioned at similar rates. $, significant difference between HC and LC rats (all *p* values < 0.0001); &, significant difference of Day 1 versus Day 2 and Day 3 (all *p* values < 0.0001). Air HC, *n* = 6; AIE HC, *n* = 9; Air LC, *n* = 12; AIE LC, *n* = 9.

Taken together, the above results suggest that AIE exposed male, but not female, adult rats exhibit significant alterations in freezing behavior associated with conditioned fear learning. However, the results also revealed that female rats as a group exhibited significantly lower levels of freezing compared to male rats. We therefore examined whether the observed lack of effect of AIE on fear and extinction behaviors in female rats might have been associated with lower levels of fear acquisition exhibited by some of the female rats compared to male rats. To test this, we separated the female rats into two groups based upon their level of freezing during fear conditioning. A cutoff of 73.3% freezing was used for classification as either a high conditioning (HC) or low conditioning (LC) rat. This classification criteria represented one standard deviation below the average percent freezing observed on conditioning on days 2 and 3 of the male rats. None of the male rats fell below this cutoff, and thus all male rats were considered to be high conditioners. The BEC levels for the HC group were 287 ± 19.7 mg/dl and 253 ± 15.85 mg/dl for the LC group. Unpaired t-test analysis revealed these were not significantly different (*p* = 0.0789). As shown in Figure 6A, comparison of freezing at Conditioning Day 3 for the HC and LC female rats revealed a significant group difference (F (1, 32) = 46.17, *p* < 0.0001). Post-hoc comparisons confirmed the level of freezing by both the Air and AIE HC rats was significantly higher than freezing by the Air and AIE LC rats (all *p* values < 0.001). When examining the time-course of fear conditioning across days, the HC group of female rats acquired fear conditioning at a similar rate to the male rats (compare Figure 6B with Figure 2A). ANOVA revealed a significant main effect of Conditioning Day (F (2, 39) = 177.3, *p* < 0.0001). Post-hoc analysis further indicated the level of freezing for both Air and AIE rats on Conditioning Day 1 was significantly different from freezing on Conditioning Day 2 and 3 (all *p* values < 0.0001). In contrast, while ANOVA of the LC female rats (Figure 6C) revealed a significant main effect of Conditioning Day (F (2, 57) = 8.825, *p* = 0.0005), post-hoc analysis indicated there were no significant differences across any of the conditioning days. ANOVA also indicated that AIE had no effect on freezing in either the HC (F (1, 39) = 1.116, *p* = 0.2973) or LC (F (1, 57) = 1.881, *p* = 0.1756) rats.

**FIGURE 7:**
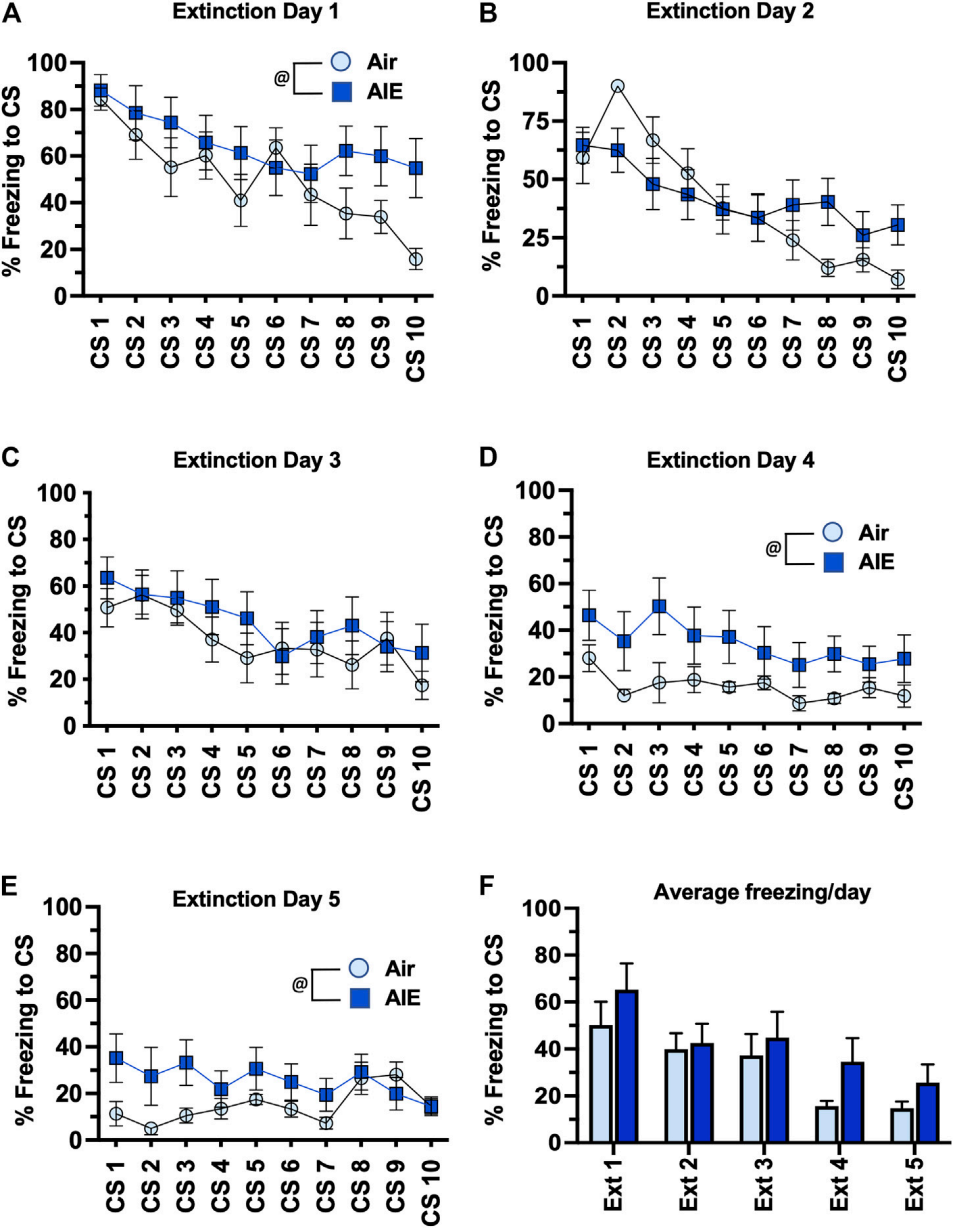
AIE exposure did not alter extinction of conditioned fear in high conditioning (HC) female rats. **(A–E)** Five consecutive days of presentation of the CS (tone) without the shock (10/session/day) resulted in a day and session-dependent reduction of freezing to presentation of the tone. While AIE exposure was associated with small but statistically significant alterations freezing on several of the days of extinction, analysis of the average freezing across the 10 tones for each daily extinction session **(F)** indicated there were no significant differences between the Air control and AIE exposed rats at any of the 5 extinction days. @, significant main effect of AIE exposure (all *p* values < 0.01). Air, *n* = 6; AIE, *n* = 9.

**FIGURE 8:**
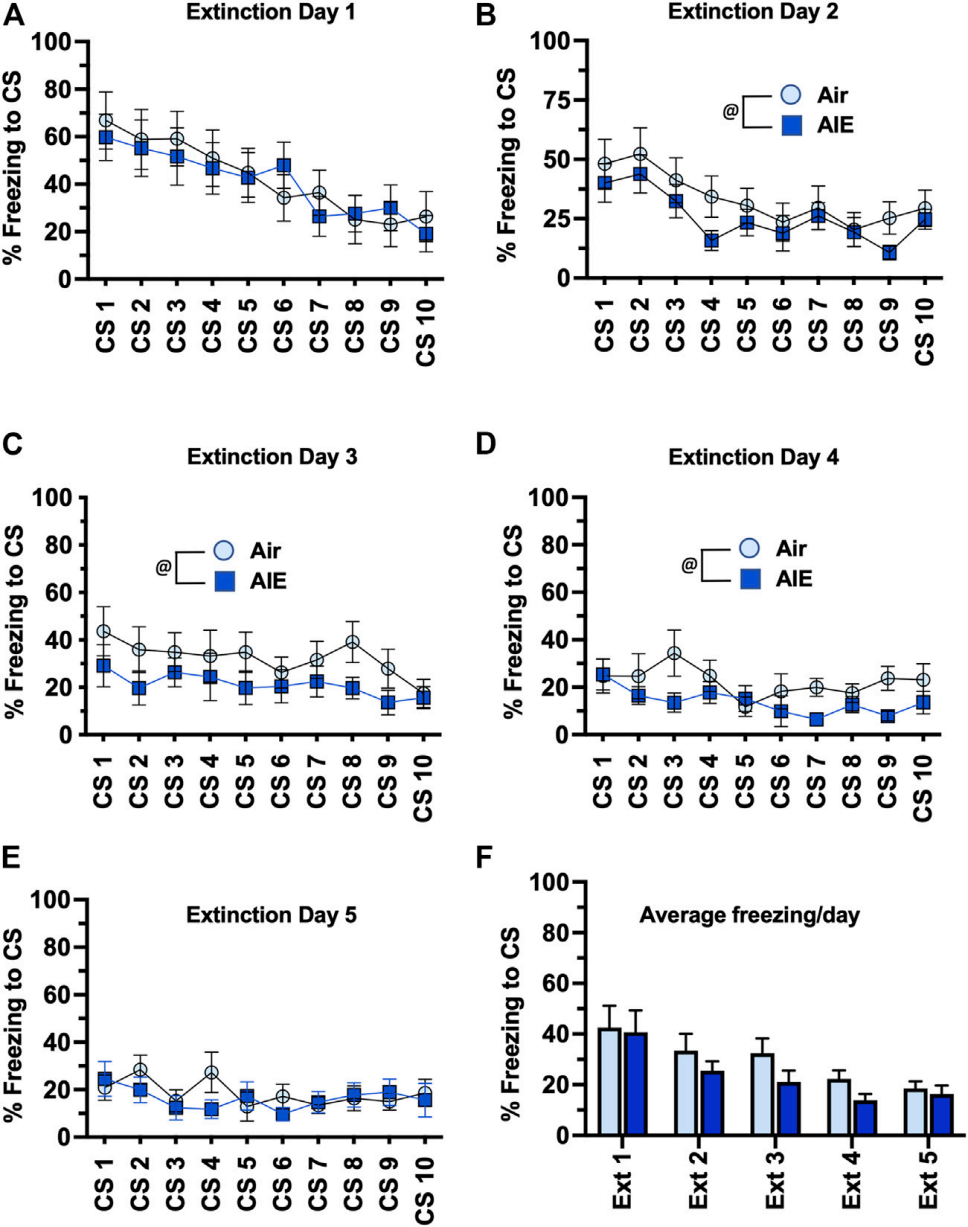
AIE exposure did not alter extinction of conditioned fear in low conditioning (LC) female rats. **(A–E)** Five consecutive days of presentation of the CS (tone) without the shock (10/session/day) resulted a session-dependent reduction of freezing to presentation of the tone. While AIE exposure was associated with small but statistically significantly changes in freezing behavior on several of the extinction days, comparison of the average freezing across the 10 tones of each daily extinction session **(F)** revealed there were no significant differences between the Air control and AIE exposed rats. Air, *n* = 6; AIE, *n* = 9. @, significant main effect of AIE exposure (all *p* values < 0.05). Air, *n* = 12; AIE, *n* = 9.

We next compared freezing in the HC and LC rats across the 5 days of extinction training. With the HC group of female rats (Figures 7A–E), ANOVA revealed a significant effect of CS# at Extinction Day 1 (F (9, 130) = 3.418, *p* = 0.0008) and Day 2 (F (9, 130) = 7.664, *p* < 0.0001), but no significant main effect of CS# at Extinction Days 3–5. There was also a significant increase in freezing in the AIE rats compared to the Air control rats at Extinction Day 1, 4 and 5 (Day 1: F (1, 130) = 8.739, *p* = 0.0037; Day 4: F (1, 130) = 18.92, *p* < 0.0001; Day 5: F (1, 130) = 9.542, *p* = 0.0025). When comparing the average freezing across each extinction day (Figure 7F), ANOVA revealed there was a significant effect of Extinction Day (F (4, 65) = 4.991, *p* = 0.0014). However, while there was a trend towards an increase in freezing in the AIE HC rats compared to the Air HC rats, this was not a statistically significant effect (*p* = 0.069). Analysis of freezing across the 5 days of extinction in the LC female rats (Figures 8A–E) revealed a significant effect of CS# at Extinction Day 1 (F (9, 190) = 3.604, *p* = 0.0004) and Day 2 (F (9, 190) = 3.220, *p* = 0.0012), but no significant main effect of CS# at Extinction Days 3–5. ANOVA also revealed there was a significant decrease in freezing in the AIE LC rats compared to the Air control LC rats at Extinction Day 2, 3 and 4 (Day 2: F (1, 190) = 4.883, *p* = 0.0283; Day 3: F (1, 190) = 9.553, *p* = 0.0023; Day 4: F (1, 190) = 9.271, *p* = 0.0027). When comparing the average freezing across the time-course of extinction training (Figure 8F), there was a significant effect of Extinction Day (F (4, 95) = 6.086, *p* = 0.0002), but no effect of treatment (Air vs AIE) (F (1, 95) = 3.148, *p* = 0.0792).

**FIGURE 9:**
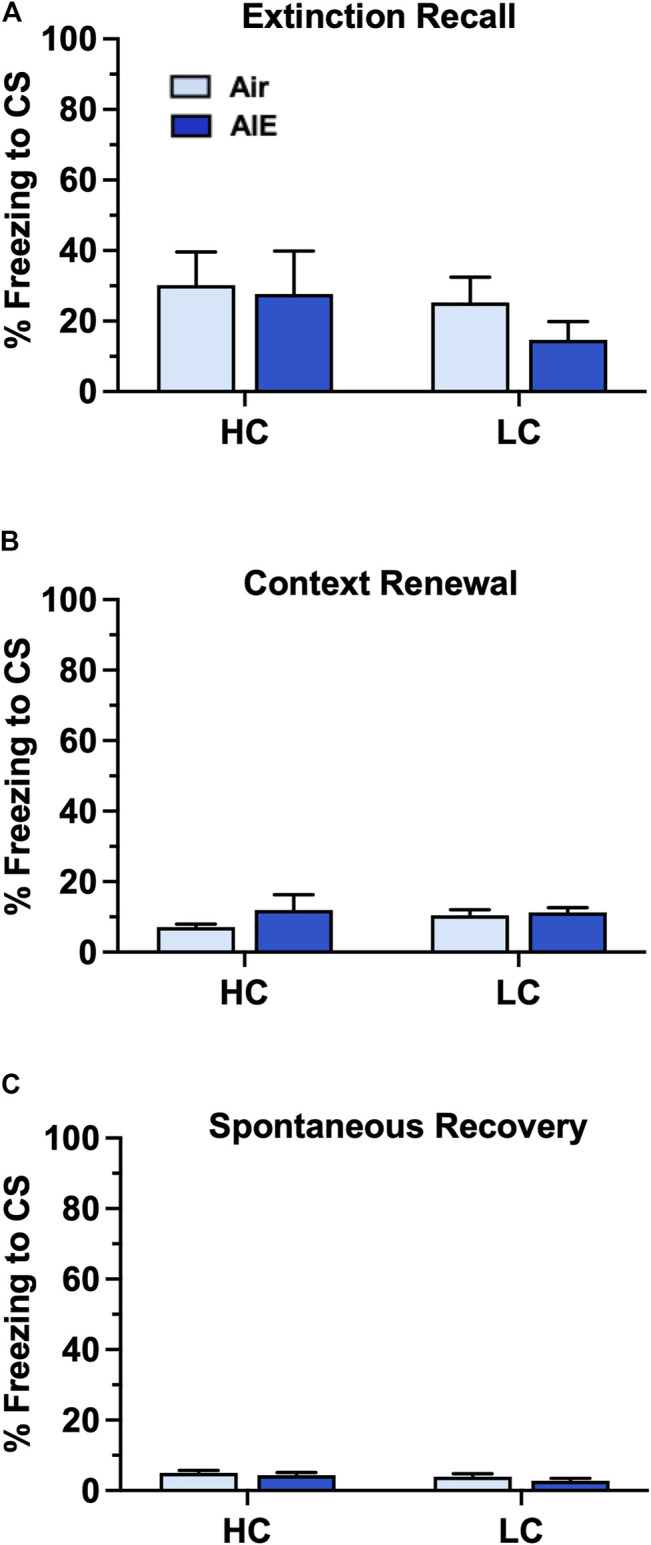
AIE exposure did not alter fear recall, renewal or spontaneous recovery in either high conditioning (HC) or low conditioning (LC) female rats. **(A)** Following 5 days of extinction training, Air control and AIE exposed adult rats were tested for extinction recall by measuring freezing behavior in response to exposure to a single conditioned tone. This revealed that HC and LC rats exhibited similar levels of freezing during recall, and that AIE exposure had no effect on recall in either HC or LC rats. **(B)** Following testing of recall, rats were tested for contextual renewal by measuring freezing behavior in response to re-exposure to the fear conditioning context. This revealed that HC and LC rats exhibited similar levels of contextual renewal, and that AIE exposure had no effect on freezing in either group of rats. **(C)** Rats were tested for spontaneous recovery 21 days after the last extinction session by placing them back into the extinction environment. This revealed that HC and LC groups exhibited minimal spontaneous recovery of the extinction memory and that AIE exposure had no effect on freezing in either group of rats. Air HC, *n* = 6; AIE HC, *n* = 9; Air LC, *n* = 12; AIE LC, *n* = 9.

We next examined the level of freezing by HC and LC rats in the tests of fear recovery that followed extinction training. As shown in Figures 9A–C, this analysis indicated there was no significant main effect of group (HC vs LC) or treatment (Air vs AIE) with Recall (Group: F (1, 32) = 0.9783, *p* = 0.3300; Treatment: F (1, 32) = 0.5235, *p* = 0.4746 ), Context Renewal (Group: F (1, 32) = 0.2455, *p* = 0.6237; Treatment: F (1, 32) = 1.198, *p* = 0.2818), or Spontaneous Recovery (Group: F (1, 32) = 2.565, *p* = 0.1191; Treatment: F (1, 32) = 1.579, *p* = 0.2180). Taken together, the above analysis suggests that the significantly lower level of fear conditioning exhibited by a subgroup of female rats did not appear to underlie the absence of an AIE effect on measures of fear recovery in females.

**FIGURE 10:**
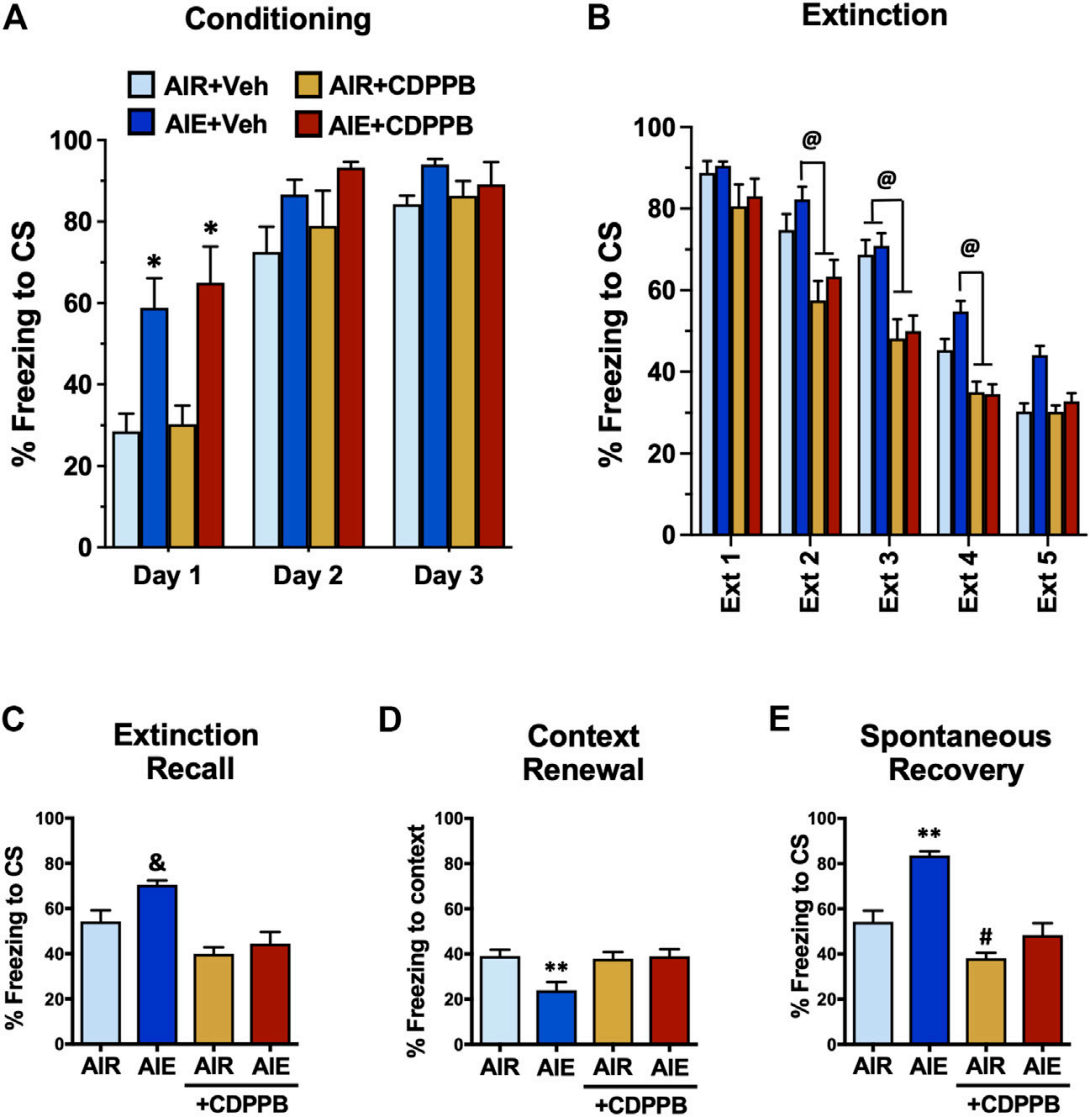
Positive allosteric modulation of mGlu5 receptors reversed AIE-induced alterations of conditioned fear behaviors in adult male rats. **(A)** Prior to administration of the mGluR5 positive allosteric modulator CDPPB during extinction training, all rats underwent 3 days of conditioning. Only rats that had been exposed to AIE exhibited significantly greater freezing during conditioning. **(B)** Administration of CDPPB 20 min prior to each extinction session resulted in significant reductions in freezing on Extinction Day 2–4. Subsequent assessment of recall **(C)**, renewal **(D)**, and spontaneous recovery **(E)** revealed that CDPPB administration during extinction training prevented AIE-induced alterations freezing in all three tests of fear responding. *, significantly different from Day 1 Air + vehicle and Air + CDPPB groups, all *p* values < 0.05; @, significantly different from Air + CDPPB and AIR + CDPPB groups, *p* < 0.05; &, significantly different from both CDPPB groups, all *p* values < 0.001; **, significantly different from all other groups, all *p* values < 0.05; #, significantly different from AIE group, *p* = 0.0487.

We have previously shown that administration of the mGlu5 PAM CDPPB can reverse AIE-induced deficits in behavioral flexibility. Therefore, we performed a final set of experiments to determine if administration of CDPPB prior to each extinction session could prevent AIE-induced alterations of fear conditioning and recovery that was observed in male AIE exposed rats. These studies were conducted only in male rats since AIE did not alter fear behaviors in the female rats. As shown in Figure 10A, a three-way ANOVA revealed a significant main effect of Conditioning Day (F (2, 60) = 73.93, *p* < 0.0001), AIE (F (1, 60) = 31.93, *p* < 0.0001), and CDPPB (F (2, 60) = 6.146, *p* = 0.0037). Post-hoc comparisons further indicated that on Conditioning Day 1, the AIE and AIE+CDPPB rats froze significantly more than all other groups of rats (all *p* values < 0.05). When comparing the average freezing on each day of extinction training (Figure 10B), a three-way ANOVA confirmed there was a main effect of Extinction Day (F (4, 180) = 151.9, *p* < 0.0001), AIE (F (1, 180) = 9.857, *p* = 0.0020), and CDPPB (F (1, 180) = 81.55, *p* < 0.0001). Post-hoc analysis indicated that CDPPB significantly reduced freezing behavior compare to the Air control and/or AIE rats at Extinction Days 2–4 (all *p* values < 0.05).

Following completion of extinction training, the rats were subjected to our standard method of testing for fear recovery. For recall (Figure 10C), a one-way ANOVA indicated a significant group difference (F (3, 20) = 11.69, *p* = 0.0001). Post-hoc analysis further revealed that both groups of rats that received CDPPB froze significantly less compared to AIE exposed rats (all *p* values < 0.001). While there was a trend towards an effect of AIE compared to the Air control rats (*p* = 0.0505), this just missed the minimum criteria to be considered a statistically significant effect. Analysis of contextual renewal (Figure 10D) also showed a significant group difference (F (3, 20) = 5.310, *p* = 0.0074). Post-hoc analysis indicated that AIE exposure resulted in a significant reduction in freezing compared to all other groups, and that this reduction was absent in the CDPPB treated rats (all *p* values < 0.05). For spontaneous recovery (Figure 10E), ANOVA again revealed a significant group difference (F (3, 20) = 25.29, *p* < 0.0001). Post-hoc analysis indicated that AIE exposed rats froze significantly more compared to all other groups, and that the AIE-induced increase was absent in the CDPPB group (all *p* values < 0.0002). Post-hoc analysis also revealed that freezing by the AIR+CDPPB rats was significantly lower compared to the Air control rats (*p* = 0.0487).

## Discussion

The present study utilized Pavlovian conditioning to examine the impact of repeated episodes of binge-like alcohol exposure during adolescence on fear learning and memory in male and female adult Long-Evans rats. The fear procedure involved a sequential ABBAB contextual design separated into three phases: an initial conditioned fear acquisition phase performed in context A, a cued-fear extinction phase performed in context B, and a fear recovery phase in which testing was performed in either the conditioning context (renewal) or extinction context (recall and spontaneous recovery). Our results revealed both sex differences and sex-specific effects of AIE on fear conditioning and fear recovery.

The conditioning procedure used consisted of a total of the 9 tone-shock pairings separated into 3 presentations per day for 3 consecutive days. This multi-day conditioning approach has an advantage over single-session conditioning as it introduces a more protracted temporal component to the acquisition of conditioned fear, which can serve as a form of context. As expected, we observed that the percent time male Air control rats spent freezing in response to tone-shock presentations increased over conditioning days, with the largest increase occurring between conditioning day 1 and 2. Male AIE exposed rats exhibited increases in freezing during tone-shock presentations compared to the male Air control rats. While this increase was observed across the 3 days of conditioning (e.g., an overall main effect of AIE), the AIE-induced increase in freezing during the tone-shock presentation was particularly prominent on conditioning day 1, which then stabilized on conditioning days 2–3 at substantially elevated levels (>80%) in both Air and AIE exposed groups. Previous studies have shown that AIE exposure is associated with increased anxiety-like behaviors in adulthood (Kyzar et al., 2019). This increase was also shown to be dependent upon AIE-induced epigenetic reprograming in the amygdala. Since the amygdala is a key brain region of the fear neurocircuitry, it is reasonable to suggest that the AIE-induced increase in fear conditioning is consistent with changes in how the amygdala processes information of potential environmental threats.

Similar to the multi-day conditioning procedure, extinction training was conducted over a 5-day period that also introduced a protracted temporal component to extinction learning. We observed that male Air control rats displayed both within-session and between-session reductions in the percentage of time spent freezing in response to CS presentation. This observation is consistent with previous studies that also utilized a protracted extinction protocol (Elias et al., 2010; Smiley et al., 2020; Smiley et al., 2021a). While some within session differences in freezing between the Air control and AIE exposed rats were noted, there was no significant difference in extinction of conditioned freezing when examined across days of extinction training.

While the neurocircuitry of fear and extinction is complex, several brain areas are thought to play particularly important roles in this neuronal network. The basolateral amygdala (BLA) functions as a central hub region where activity-dependent plasticity critically shapes fear learning and memory (Ciocchi et al., 2010; Amano et al., 2011). Inputs from the ventral hippocampus (vHP) to the BLA provides contextual information of the threat that is critical for the expression of conditioned fear, while subregions of the mPFC differentially regulate fear conditioning and extinction. In particular, excitatory inputs from the prelimbic (PrL) sub-region to the BLA promote the expression of conditioned fear whereas inputs from the infralimbic (IfL) sub-region promote fear extinction (Tovote et al., 2015; Day and Stevenson, 2020). Previous reports that AIE exposure leads to deficits in behavioral flexibility (Spear, 2018), including resistance to the extinction of ethanol self-administration (Gass et al., 2014b), lead us to hypothesize that AIE exposure might also be associated with resistance to extinction of conditioned fear. Adding further support to this suggestion, the enhanced freezing we observed in AIE exposed rats during fear conditioning might also be expected to lead to differences in fear extinction, especially during the initial time-course of extinction training. However, our observations that male AIE exposed rats extinguished conditioned freezing at a similar rate and extent compared to male Air rats did not support this idea.

An interesting observation in the present study was that male AIE exposed rats exhibited significant alterations in freezing on all three tests of fear recovery in comparison to the male Air control rats. The AIE-induced alterations in fear recovery included increased freezing during fear recall and spontaneous recovery, and reductions in freezing during fear renewal. Therefore, in contrast to a lack of effect of AIE on fear extinction, AIE exposed male rats exhibited significant alterations during assessment of post-extinction fear memory. The reduction in recall (observed as an increase in freezing) appeared to represent a protracted change since an increase in freezing was also observed during spontaneous recovery assessed 21 days after extinction training. A previous study conducted in male Sprague-Dawley rats also reported that intermittent ethanol exposure during early adolescence disrupted fear conditioning in adulthood (Broadwater and Spear, 2013). While that study did not observed an effect of AIE on the acquisition of condition fear, they did report reductions of contextual renewal similar to the present study. A number of previous studies, mostly conducted in male rats, have shown that repeated episodes of binge-like adolescent alcohol exposure leads to deficits in behavior control in adulthood (Spear, 2018). Therefore, given the overlap of the neurocircuitry of fear (e.g., mPFC, HP, BLA) with the neurocircuitry of cognitive and emotional behaviors, it is not surprising that AIE-induced alterations in anxiety and cognition also extend to long-term alterations in threat responding. Adolescence is a period during which threat monitoring/response and the neurocircuits that mediate this behavior are highly plastic and undergoing rapid changes (Gerhard et al., 2021), and emotional experiences during this time can profoundly shape long-term functioning of the neurocircuitry that mediates affective behavior. Thus, our observation may have important translational implications as difficulties in assessing and appropriately responding to potential threats is a key feature of many psychiatric disorders that may be impacted by a history of adolescent alcohol abuse.

Another interesting observation in the present study was the striking differences between male and female rats in fear conditioning and recovery. In humans, the vulnerability to develop fear and anxiety-related disorders is twice as high in females compared to males (Day and Stevenson, 2020). However, results from studies investigating sex differences in rodent are less clear. While studies of Pavlovian fear learning suggest that female rodents typically exhibit reductions in contextual fear conditioning (Maren et al., 1994; Pryce et al., 1999; Wiltgen et al., 2001; Kosten et al., 2005; Gresack et al., 2009; Ribeiro et al., 2010), the results of fear extinction have been mixed (Velasco et al., 2019). Regardless, a number of studies have reported reduced contextual fear conditioning in females compared to males as evidenced by reductions in freezing during later retrieval tests of contextual fear memory. In the present study, female rats exhibited significant reductions in conditioned freezing compared to males, more rapid extinction of freezing, lower levels of freezing during recall and contextual renewal, and a near complete absence of freezing during spontaneous recovery. The latter observation of the reduction in spontaneous recovery in female rats appears to be in contrast to a previous report that females exhibit increases in spontaneous recovery (Fenton et al., 2014). However, considerable methodological and procedural differences between the two studies caution against a direct comparison of the results. For the most part, our observations are in general agreement with the studies showing female rats exhibit reduced acquisition of conditioned freezing and contextual fear retention compared to males. They are also in agreement with a recent study showing that AIE exposed male mice exhibited enhanced contextual fear acquisition compared to the male Air controls (Kasten et al., 2020). This effect was sex-specific as AIE did not alter fear acquisition in female mice. To ascertain whether the lower level of fear conditioning observed in the female rats may have contributed to an apparent reduction in fear extinction and responding, we classified female rats as either high conditioning subjects or low conditioning subjects. This analysis revealed that both the high and low conditioning groups of female rats displayed similarly low levels of freezing during test of recall, renewal and spontaneous recovery.

It is worth noting that caution should be exercised when interpreting results of sex differences based solely on freezing behavior. The amount of time an animal spends freezing during the CS is typically interpreted to reflect the strength of the tone-shock association, with low freezing reflecting a weak associative memory. However, female rats have been reported to be more active than male rats and may adopt more active avoidance strategies compared to less active male rats (Beatty and Beatty, 1970; Beatty and Fessler, 1976; Hyde and Jerussi, 1983; Steenbergen et al., 1990; Fernandes et al., 1999; File, 2001). Consistent with this, a novel form of active fear avoidance—termed “darting”—has been observed in females rats during fear conditioning (Gruene et al., 2015; Colom-Lapetina et al., 2019). Animals that display this form of coping behavior are also less likely to freeze in response to the tone, leading to the suggestion that darting behavior in females may represent an active coping response (Gruene et al., 2015; Jones and Monfils, 2016). Furthermore, these animals also exhibited more rapid reductions in both darting and freezing behaviors during extinction, and thus darting may be a form of active coping that promotes long-term reductions in fear. Studies have also shown that female rats tend to be more risk aversive than male rats and display greater active avoidance (Orsini et al., 2016; Orsini et al., 2021), which may contribute to greater vulnerability to anxiety-related disorders in women compared to men (Li and Graham, 2017).

Another caveat to interpretation of our data relates to potential differences in sensitivity between males and females to foot shock. As with most studies that compare fear conditioning in males and females, we used the same shock intensity (0.75 mA) during conditioning in both sexes. While we cannot rule out that differences in sensitivity to the shock contributed to our observations of sex difference in fear conditioning and recovery, we believe it is unlikely to have played a significant role. In fact, female rats have been reported to be more sensitive, not less sensitive, to shock than males (Beatty and Fessler, 1976; Kosten et al., 2005; Dalla and Shors, 2009).

In contrast to observations in adult male rats, adult AIE exposed female rats did not exhibit differences in freezing compared to the Air control females during of fear conditioning, extinction, or fear recovery. In our AIE procedure, we quantify the level of behavioral intoxication immediately upon removal of the animals from the chambers, and then use that information to guide subsequent dosing adjustments in the amount of alcohol vapor delivered to the chambers during the next exposure cycle. This approach resulted in only slightly different levels of intoxication in the female rats compared to the male rats (2.4 ± 0.09 for female rats versus 2.66 ± 0.16 for male rats on the intoxication rating scale). However, the corresponding blood ethanol levels in the females were significantly lower than the levels in males (271 ± 12.7 mg/dl for the female rats versus 383 ± 9.6 mg/dl for the male rats). While the lower levels of blood ethanol in the female rats could in theory have differentially impacted fear conditioning in adulthood, it should be noted that these blood ethanol levels are relatively high and both model extreme binge intoxication in humans.

As noted above, AIE exposure results in impairments in behavioral flexibility and cognitive control in the adults. We have also previously demonstrated that administration of CDPPB, a PAM of mGlu5 receptors with pro-cognitive actions, can reverse these deficits (Gass et al., 2014a). In the present study, we extended these observations to threat responding by demonstrating that PAM of mGlu5 receptors completely prevented the alterations in fear recovery in the male AIE exposed rats. Importantly, while CDPPB was administered only during fear extinction training, its effect on fear recovery was long-lasting as it normalized the subsequent AIE-induced alterations in fear recall, renewal, and spontaneous recovery. This observation may have important clinical implications for pharmacological treatments aimed at preventing the re-emergence of maladaptive fear and intrusive memories that are problematic in many psychiatric disorders. Due to the overlapping neurocircuitry associated with AUD and trauma-related disorders such as PTSD, current behavioral therapies utilize basic learning and memory principles (e.g., extinction-based exposure therapy). While they have been used as a treatment for both PTSD (Kessler et al., 1995) and drug addiction (Conklin and Tiffany, 2002), they have shown limited efficacy. The results from these studies suggest that a combination of behavioral and pharmacological treatment may be a more effective therapy for individuals who suffer from these debilitating disorders.

## Data Availability

The raw data supporting the conclusions of this article will be made available by the authors, without undue reservation.
